# Environmental factors related to differences in the microbiota in the upper respiratory tract in young children: Focusing on the impact of early nursery attendance

**DOI:** 10.3389/fped.2023.1015872

**Published:** 2023-01-30

**Authors:** Asmaa Abushawish, Kaoru Haro, Takayuki Hoshina, Naoko Kitajima, Koichi Kusuhara

**Affiliations:** ^1^ Department of Pediatrics, School of Medicine, University of Occupational and Environmental Health, Kitakyushu, Japan; ^2^ Department of Pediatrics, Sato Children’s Clinic, Kitakyushu, Japan; ^3^ Department of Pediatrics, Onga Nakama Medical Association, Onga Hospital, Onga, Japan

**Keywords:** microbiota, upper respiratory tract, nursery attendance, antibiotics, clone library analysis

## Abstract

**Background:**

Microbial colonization of the upper respiratory tract (URT) during the first years of life differs significantly according to environmental factors. We investigated the association between early nursery attendance, URT infection (URTI) and drugs used for its treatment and the differences in the URT microbiota.

**Methods:**

This prospective study included 33 young children (11 and 22 with and without nursery attendance during their infancy, respectively). URT secretions were collected from the nasopharynx of these children at 2, 4, 6, 12, 18 and 24 months old. Clinical information after the latest sampling, including histories of URTI and the uses of antibiotics or cold medicines, was collected from all children. URT bacteria were identified by a clone library analysis of the 16S rRNA gene.

**Results:**

In the diversity of URT microbiota using the Shannon index, we did not detect any associations between variations in the URT microbiota and environmental factors (nursery attendance, development of URTIs, or the uses of antibiotics or cold medicines). However, in a clustering analysis, the proportion of the samples classified as *Corynebacterium propinquum*-dominant cluster was significantly lower in children ≥6 months old with nursery attendance than in those without nursery attendance. In addition, the URT microbiota was significantly different between samples from children ≥6 months old with and without a history of ≥3 URTI episodes after the first sampling. Furthermore, the URT microbiota was also significantly different between samples from these children with and without antibiotic use between the previous and present samplings.

**Conclusion:**

Early nursery attendance and its related factors, including the frequency of URTI and antibiotic treatment, may be associated with the differences in the URT flora in young children.

## Introduction

The human body is colonized by large groups of microbes that constitutes a complex ecosystem. The composition and function of these microbial populations vary among different sites in the human body (1, 2). Many studies have reported that the microbiota determine the health of the human body and are required for the normal development of organs, such as the gut, lungs, and brain (3, 4). Like other sites in the human body, the upper respiratory tract (URT) is colonized by a large variety of bacteria constituting the normal microbiota immediately after birth, which play a protective role against pathogen invasion. Pathogenic bacteria, including *Streptococcus pneumoniae*, *Haemophilus influenzae* and *Moraxella catarrhalis*, also asymptomatically colonize the URT microbiota and occasionally have the potential to cause disease (4).

Microbial colonization of the URT during the first years of life reportedly differs significantly according to internal factors, such as genetic predisposition and external factors, including environmental factors, mode of delivery (vaginal delivery or cesarean section) and type of feeding (5–7). In recent years, the number of children who start nursery attendance from infancy has been increasing in Japan because of the trend toward an increased incidence of nuclear families and double-income household. It is considered that day-care attendance is related to an increased frequency of infectious diseases (8). Furthermore, antibiotics are frequently prescribed for children with acute respiratory tract infections (9). There have been a few studies indicating that day-care attendance was associated with a change in the URT microbiota and the colonization of *S. pneumoniae* (10, 11). However, the direct factors that change the URT microbiota in children attending day-care remain unclear. We hypothesized that the URT microbiota may be different between children with and without nursery attendance during infancy because the frequencies of the prescription of medicines and URTI are predicted to be different between these 2 populations.

In this study, we investigated the association between early nursery attendance, and drugs often prescribed for infectious diseases and the differences in the URT microbiota.

## Materials and methods

### Study population and sample collection

This prospective study included 33 children, of whom 11 (33.3%) entered the nursery school affiliated with the University of Occupational and Environmental Health, Japan at <12 months old, while 22 (66.7%) were not sent to any nursery schools during their early infancy (<12 months old). The nursery school subjects were recruited just before nursery attendance, while the other subjects were recruited when they visited either Onga Hospital or Sato Children's clinic to receive vaccines. The enrollment of the subjects was performed from 2015 to 2020. All children completed four doses of the 13-valent pneumococcal conjugate vaccine and *H. influenzae* type b conjugate vaccine. When the subjects were enrolled in this study, we obtained the demographic information of each child including their gender, gestational-age, birth weight, underlying diseases and family history. The clinical information after their latest sampling, including histories of URT infection (URTI) and the usage of antibiotics and cold medicines, including antitussive agents, expectorant drugs and sinus medicine, was collected from all children. URT secretions were collected from the nasopharynx of children using nasal swabs at 2, 4, 6, 12, 18, and 24 months old, and then were suspended in 1 ml of phosphate-buffered saline just after the sample collection. The samples were stored at −20°C for the bacterial gene analysis. Informed consent was obtained from the parents of the children.

### Total bacterial cell counts and cell lysis efficiency analyses

Part of the suspension (100 µl) from the nasal swab sample was added to 900 µl of ethidium bromide solution [100 µg/ml in 0.1 M phosphate buffer (pH 8.5), 5% NaCl, 0.5 mM ethylenediaminetetraacetic acid-2Na]. After vortexing the mixture (1.0 ml), it was left at room temperature for 10 min, and filtered through a 0.2-µm-pore filter (Millipore, Bedford, MA, United States). Subsequently, the filter was placed on a glass slide, and one drop of glycerine was inserted before covering the glass. The number of bacteria was counted in 30 randomly selected fields using an Olympus BX40 epifluorescence microscope (Olympus Optical, Tokyo, Japan), and the number of bacteria per millilitre of solution was then calculated. The same method was used to count the remaining bacteria numbers after DNA extraction treatment in order to check the cell lysis efficiency. The cell lysis efficiency was calculated as the ratio of the number of bacteria remaining after the DNA extraction treatment to the total number before the treatment [100 - (post-extraction number/pre-extraction number) × 100]. We judged the samples to be appropriate for further analyses when the cell lysis efficiency was >80% (12). No further analyses were performed for samples that did not reach an efficiency of 80%.

### DNA extraction

Vigorous shaking of the suspension from the nasal swab together with sodium dodecyl sulfate solution n (final concentration, 3.0%) and glass beads was used to extract DNA, as previously reported (13).

### Polymerase chain reaction (PCR) of the 16s rRNA gene

After DNA extraction, the partial 16S rRNA gene fragments were amplified *via* PCR using a Veriti thermocycler (Applied Biosystems, Foster City, CA, USA). Reaction mixtures containing the universal 16S rRNA gene primer set (E341F:5′-CCTACGGGAGG-CAGCAG-3′ and E907R: 5′-CCGTCAATTCMTTTRAGTTT-3′) and AmpliTaq Gold DNA polymerase LD (Applied Biosystems) were incubated in a thermocycler at 96°C for 5 min, followed by 30 cycles at 96°C for 30 s, 53°C for 30 s, and 72°C for 1 min and then 1 cycle for the final elongation step at 72°C for 7 min.

### Clone library construction and determining of nucleotide sequences

The amplified PCR products were cloned into *Escherichia coli* using a TOPO TA cloning kit (Invitrogen, Carlsbad, CA, USA), and then the nucleotide sequences of 96 randomly selected colonies were determined from each clone library for a sequencing analysis. Subsequently, we amplified the partial fragments of the cloning vectors (pCR 4) with AmpliTaq 360 Master Mix, GC Enhancer and a Pre-Seq primer set (F; 5′-GTTTTCCCAGTCACGACG-3′ and R; 5′- CAGGGAACAGCTATGAC-3′; Applied Biosystems). An Exonuclease I and Alkaline Phosphatase (Shrimp) (TaKaRa Bio Inc., Otsu, Shiga, Japan) was used to eliminate the primers and deoxyribonucleotided triphosphate from the PCR mixture in a clean-up process. From that, a 1 µl aliquot was used as a template for the sequencing reaction to perform with primers M13 F and the BigDye Terminator Cycle Sequencing Kit v3.1 (Applied Biosystems). The nucleic acid sequences were determined on a 3130xl Genetic Analyzer (Applied Biosystems) as the final of the process.

### Homology search

The Geneious prime software program (Biomatters Ltd., Auckland, New Zealand) was used to check the quality and trimming of the sequences. Approximately 550 base pairs (bp) were screened to detect the sequences. The comparison of highly accurate detected sequences with a database containing 16s rRNA gene sequences of type strains was performed using a basic local alignment search tool algorithm (blastn). A phylotype sharing ≥97% homology with the sequence of the type strain using the basic local alignment search tool algorithm (blastn) was considered to be a presumptive species in this study. *H. aegyptius* and *H. influenzae* were described as “*H. aegyptius/influenzae*”, whereas *M. catarrhalis* and *M. nonliquefaciens* were defined as “*M. catarrhalis/nonliquefaciens*” because they cannot be distinguished by an analysis of 550-bp PCR product amplified by the universal 16S rRNA gene primer set.

### Statistical analyses

Data analyses including comparison of environmental factors were performed using the RStudio Desktop (data analysis R packages, ver. 4.0.4). The vegan package-based estimate R function was used to assess the Alpha- diversity by the Shannon index, while the heatmap. 3 package was used to create the heatmap. The BellCurve plugin for Excel, version 3.21 software (Social Survey Research Information, Tokyo, Japan) was used for the clustering analysis. The Mann–Whitney *U*-test was used to compare quantitative values, and the *χ*^2^ or Fisher's exact test was used for the qualitative analyses. *P*-values <0.05 were considered to be statistically significant.

### Ethical approval

Our study was approved by the Institutional Review Board of the University of Occupational and Environmental Health, Japan (H26–201).

## Results

### Subject characteristics

URT secretions were collected from the 33 enrolled children (19 males, 14 females) and their median age at the first sampling was 2 months old [interquartile range (IQR): 1–11]. A total of 118 samples were obtained from the 33 children. The median number of samples for each subject was 3 (IQR: 2–6). Demographic characteristics of the enrolled children and information on them at the time of sampling are shown in Table 1. The proportions of children treated with antibiotics or cold medicines and those with a total of ≥3 URTI episodes during the investigation period were not markedly different between children with and without nursery attendance (Table 2). However, the proportion of children treated with antibiotics before 12 months old was significantly higher in the nursery attendance group than in the non-nursery attendance group (*P* = 0.03).

**Table 1:** Demographic characteristics of the enrolled children and information on children at the time of sampling.

Demographic characteristics	
Number of subjects, *n*	33
Age at first sampling, months, median (IQR)	2 (1–11)
Sex, number of males, (%)	19 (57.5%)
History of admission to neonatal intensive care unit, *n* (%)	1 (3.0%)
Nursery attendance during infancy, *n* (%)	11 (33.3%)
Information on children at the time of sampling	
Number of samplings, *n*	118
Age at the sampling, months, median (IQR)	8 (2–38)
Nursery attendance, *n* (%)	41 (34.7%)
Use of antibiotics between the previous and present samplings, *n* (%)	42 (35.5%)
Use of cold medicines between the previous and present samplings, *n* (%)	44 (37.2%)
Development of upper respiratory tract infection for ≥3 times after first sampling, *n* (%)	12 (10.0%)

IQR, interquartile range.

**Table 2:** Comparisons of the demographic and clinical characteristics between children with and without nursery attendance.

	Children with nursery attendance (*n* = 11)	Children without nursery attendance (*n* = 22)	*P*-value
Sex, number of males, (%)	5 (45)	14 (64)	0.31
History of admission to NICU, *n* (%)	0	1 (5)	0.48
Use of antibiotics, *n* (%)^a^	10 (91)	19 (86)	0.71
Use of antibiotics ≤12 months, *n* (%)	10 (91)	11 (50)	0.03
Use of cold medicines, *n* (%)^a^	9 (82)	18 (82)	1.00
Use of cold medicines ≤12 months, *n* (%)	8 (72)	11 (50)	0.28
Development of URTI for a total of ≥3 times, *n* (%)^a^	2 (18)	5 (23)	0.76
Development of URTI ≤ 12 months, *n* (%)	9 (82)	18 (82)	1.00

NICU, neonatal intensive care unit; URTI, upper respiratory tract infection.

^a^ The items indicate the histories during the investigation period.

### Total bacterial numbers and cell lysis efficiency

The numbers of bacteria in URT secretions counted using epifluorescence microscopy ranged from 1.1 × 10^4^ to 1.8 × 10^9^ cells/ml (median 7.0 × 10^6^ cells/ml). The cell lysis efficiency was ≥80% in all samples (data not shown). There were no significant differences in the total numbers of bacteria between the children with and without nursery attendance, a history of URTI, or those with and without the use of antibiotics or cold medicines between the previous and present samplings.

### Diversity of the URT microbiota

**Figure 1:**
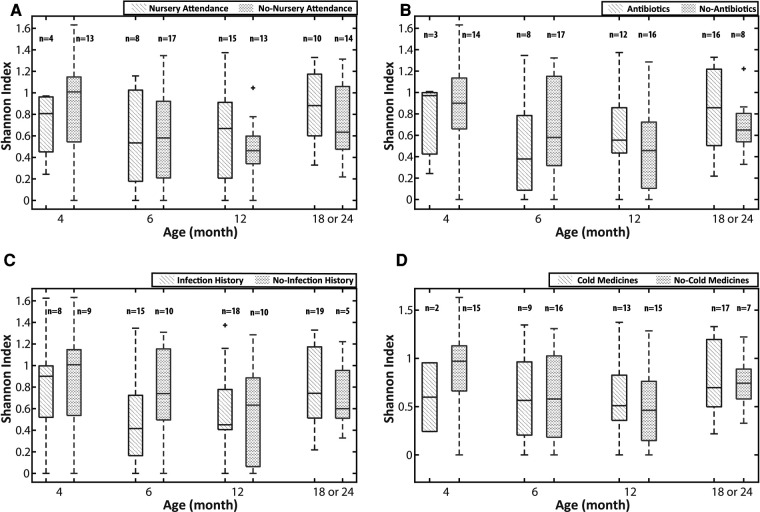
The impact of environmental factors on the diversity of the URT microbiota. (**A**) early nursery attendance, (**B**) use of antibiotics, (**C**) URTI episodes and (**D**) use of cold medicine. The Shannon index was used for the analyses of the diversity of the URT microbiota. The analyses were performed on the 4 age groups (around 4, 6, 12 and 18 or 24 months old). URTI episodes and the uses of antibiotics and cold medicines are defined as the histories between the previous and present samplings. URTI: upper respiratory tract infection, URT: upper respiratory tract.

We first analyzed the impact of early nursery attendance (entrance to nursery school at <12 months old) on the diversity of the URT microbiota of children using the Shannon index. The diversity of the URT microbiota in the samples collected from 11 children with nursery attendance before the entrance was not significantly different from that in the first samples collected from the 22 children without nursery attendance. The age of children when the first sample was collected (median 2 months old, range 1–11) did not demonstrate any significant differences among the 2 groups. Given that the URT microbiota is continuously changing during infancy, we analyzed it at 4 time points (namely at around 4, 6, 12 and 18 or 24 months old). As the number of the samples collected from children at 24 months old was small, we combined the data obtained from those who were 18 and 24 months old. In all age groups, there was not a significant difference in the diversity of URT microbiota between children with and without nursery attendance (Figure 1A). We then analyzed the impact of environmental factors related to nursery attendance on the diversity of the URT microbiota. As the development of URTI, antibiotic treatment and the drugs often prescribed for URTI are speculated to influence the differences in the URT microbiota, we selected the development of URTI and the usage of antibiotics and cold medicines as the environmental factors. In all age groups, there were no significant differences in the diversity of URT microbiota between children with and without a history of URTI or those with and without the use of antibiotics or cold medicines between the previous and present samplings (Figure 1B–D).

### Clustering analyses

**Figure 2:**
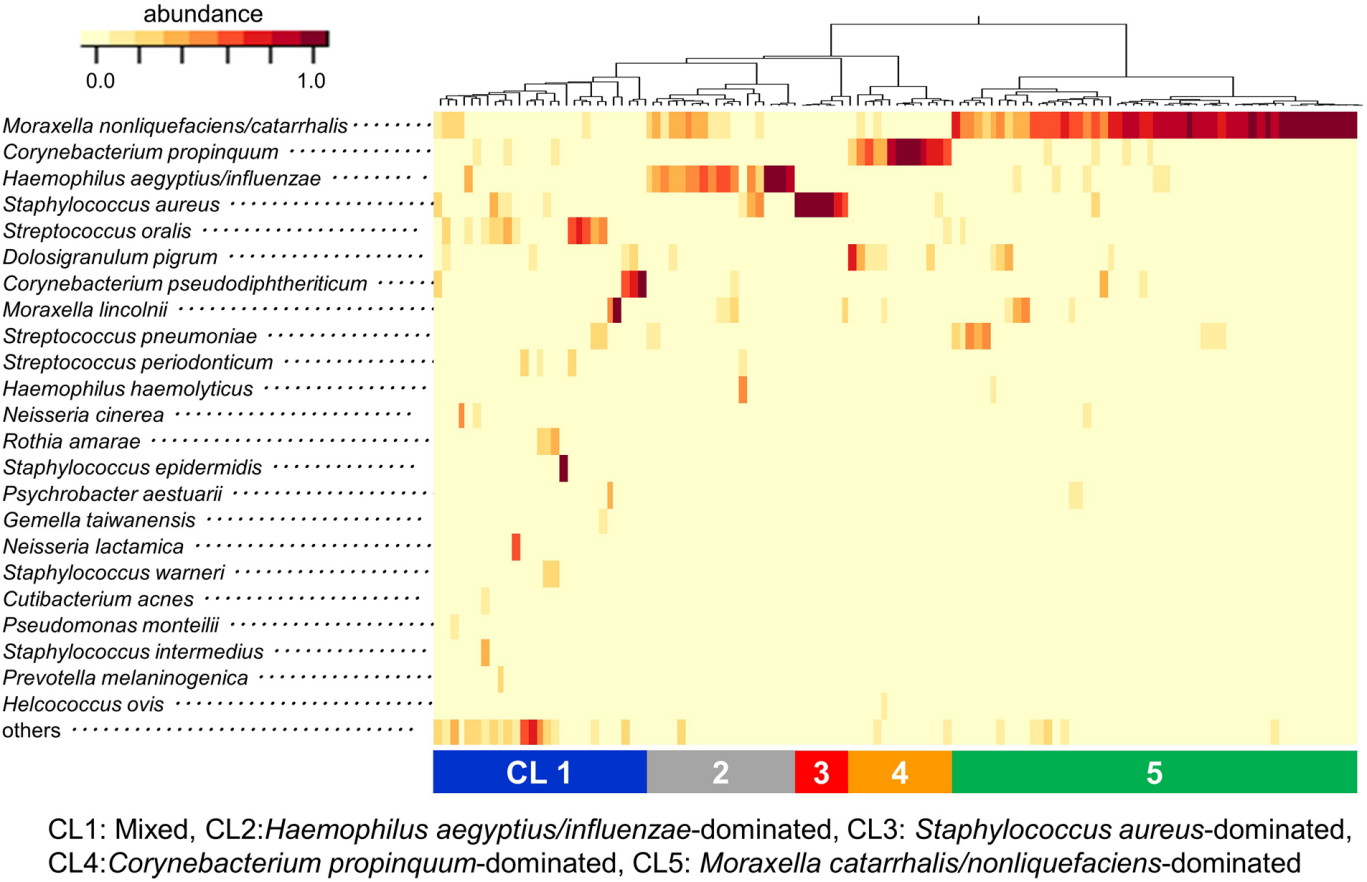
Results of clustering analysis at the species level. The relative abundance of all bacteria in each sample and the clustering results based on the composition of the bacteria are indicated by the heatmap. Clustering of samples revealed 5 distinct clusters: CL1) Mixed, CL2) *Haemophilus aegyptius/influenzae*-dominated, CL3) *Staphylococcus aureus-*dominated, CL4) *Corynebacterium propinquum*-dominated and CL5) *Moraxella catarrhalis/nonliquefaciens*-dominated. CL: cluster.

The relative abundance of all bacteria in each sample and the clustering results based on the composition of the bacteria are shown in Figure 2. The most abundant bacterium in the URT secretions was *M. nonliquefaciens/catarrhalis* (36.7%), followed by *Corynebacterium propinquum* (9.5%), *H. aegyptius/ influenzae* (9.4%), *S. aureus* (7.8%) and *Streptococcus oralis* (4.8%). The clustering of samples based on the dendrogram created by the data analysis R packages (ver. 4.0.4) and the composition of the bacteria indicated by the heatmap revealed the following 5 clusters (CLs): CL1, Mixed; CL2, *H. aegyptius/influenzae*-dominated; CL3, *S. aureus-*dominated; CL4, *C. propinquum*-dominated; CL5, *M. catarrhalis/nonliquefaciens*-dominated. All samples were then classified into these 5 clusters (Figure 2).

**Figure 3:**
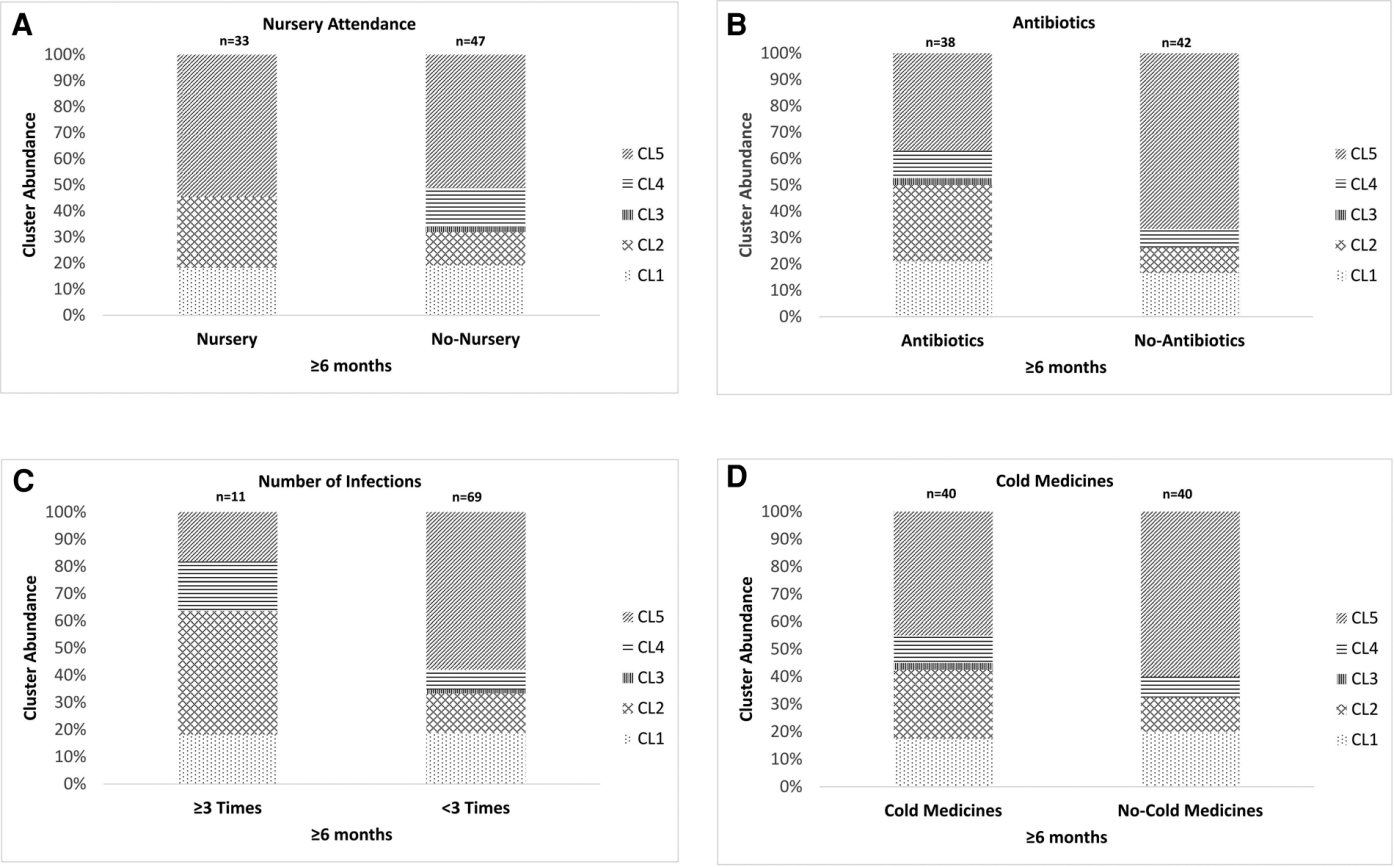
The association between the results of the clustering analysis using the URT secretions from the eligible children and the environmental factors. (**A**) Nursery attendance, (**B**) use of antibiotics after the latest sampling, (**C**) a history of ≥3 URTI episodes after the first sampling and (**D**) use of cold medicines between the previous and present samplings. The URT secretions from children ≥6 months old were used for these analyses. The 5 clusters indicate Mixed (CL1), *Haemophilus aegyptius/influenzae-*dominated (CL2), *Staphylococcus aureus-*dominated (CL3), *Corynebacterium propinquum*-dominated (CL4) and *Moraxella catarrhalis/nonliquefaciens*-dominated (CL5). Although the analyses were performed by using multiple samples collected from the same child, these samples were not always classified as the same cluster. URTI: upper respiratory tract infection, URT: upper respiratory tract, CL: cluster.

We investigated the association between the results of the clustering analysis and environmental factors. As children <6 months old had just started to attend nursery school, and their backgrounds related to nursery attendance, such as histories of the antibiotic usage and URTI, were almost the same as those of children without nursery attendance (data not shown), we could not perform any investigations in these children. We used 80 samples from children ≥6 months old consisting of 2 different populations for the investigation. The proportion of the samples classified as CL4 (*C. propinquum*-dominated cluster) was significantly higher in children without nursery attendance than in those with nursery attendance (*P* < 0.05, Figure 3A). Conversely, the proportion of the samples classified as CL2 (*H. aegyptius/influenzae*-dominated cluster) was higher in children with nursery attendance than in those without nursery attendance, although not to a significant extent. The proportion of the samples classified as CL2 was significantly higher in children with the use of antibiotics between the previous and present samplings than in those without the use of antibiotics whereas the opposite tendency was observed in the proportion of the samples classified as CL5 (*M. catarrhalis/nonliquefaciens*-dominated cluster) (*P* < 0.05, Figure 3B). The same result was shown in the comparison between samples from children with and without the development of a URTI for ≥3 times after the first sampling (*P* < 0.05, Figure 3C). The distribution of the 5 clusters was not significantly different between the samples obtained from children with and without the use of cold medicines between the previous and present samplings (Figure 3D). Although the analyses were performed by using multiple samples collected from the same child, these samples were not always classified as the same cluster.

**Figure 4:**
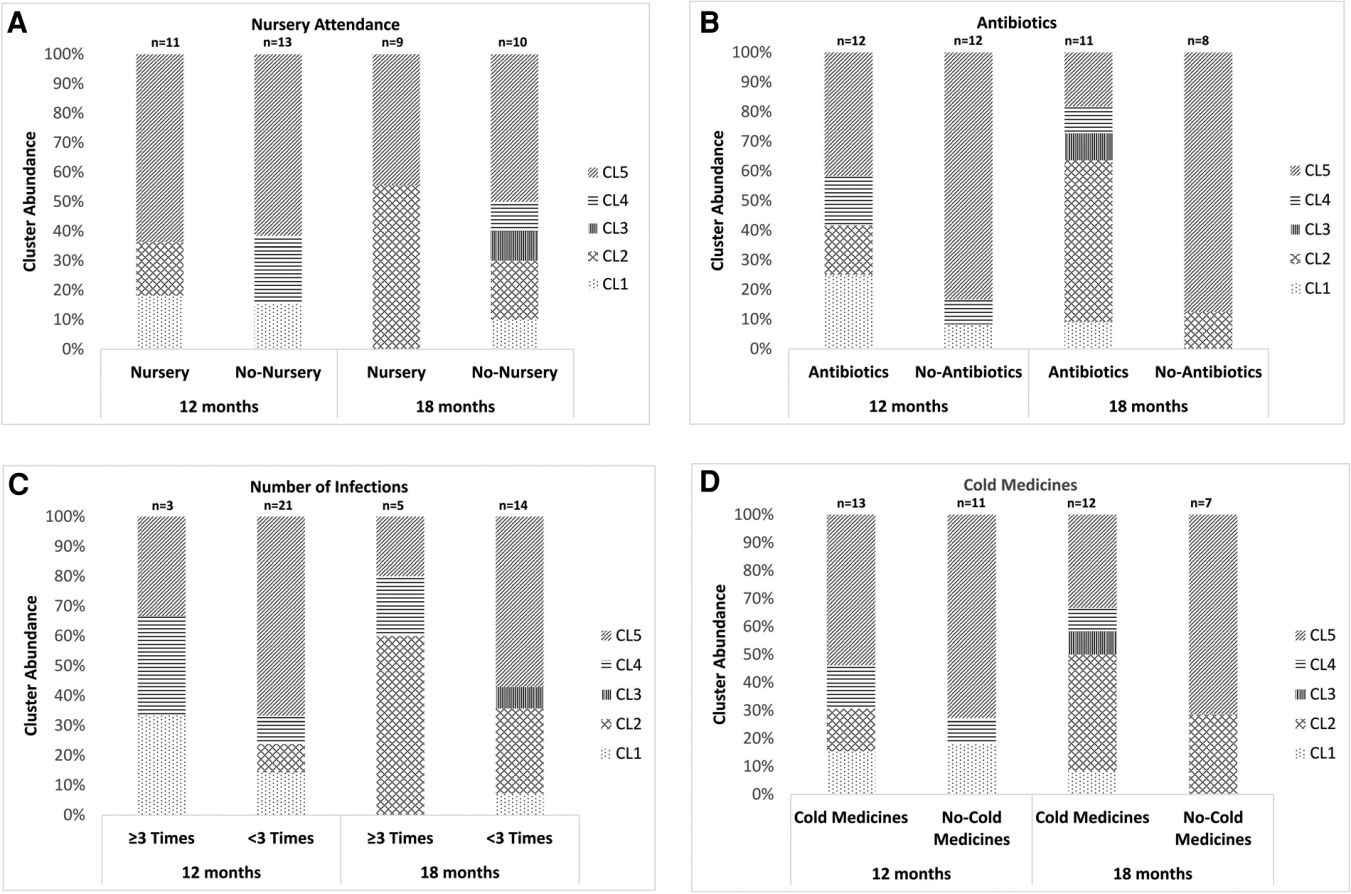
The association between the results of the clustering analysis using the URT secretions from children aged around 12 or 18 months old and environmental factors. (**A**) Nursery attendance, (**B**) use of antibiotics after the latest sampling, (**C**) history of ≥3 URTI episodes after the first sampling and (**D**) use of cold medicines between the previous and present samplings. The 5 clusters indicate Mixed (CL1), *Haemophilus aegyptius/influenzae-*dominated (CL2), *Staphylococcus aureus-*dominated (CL3), *Corynebacterium propinquum*-dominated (CL4) and *Moraxella catarrhalis/nonliquefaciens*-dominated (CL5). URTI: upper respiratory tract infection, URT: upper respiratory tract, CL: cluster.

We further performed the same analysis on 2 different age groups (children around 12 and 18 months old). An analysis of children at around 6 and 24 months old was not performed because of the small sample size. The proportion of the samples classified as CL5 was significantly lower in children at around 12 months old who had been treated with antibiotics between the previous and present samplings than in those without receiving antibiotic therapy (*P* = 0.005, Figure 4B). No other environmental factors, including nursery attendance, a history of ≥3 URTI episodes after the first sampling and the use of cold medicines between the previous and present samplings, were associated with the distribution of the 5 clusters (Figure 4A,C,D).

## Discussion

In this study, we focused on the impact of early nursery attendance on differences in the URT microbiota in young children. A previous study indicated a high abundance and low diversity of bacteria in the URT secretion from children with respiratory symptoms (14). In addition, pathogenic bacteria, including *H. influenzae* and *S. pneumoniae* more frequently colonized in the nasopharynx of children with acute respiratory infection than that of asymptomatic children (15, 16). Daycare or kindergarten attendance was associated with a significantly increased risk of both URTI and acute ear infection for children <5 years old (8). Based on these results, we hypothesized that early nursery attendance would lead to a lower diversity of bacteria in the URT microbiota and a high abundance of pathogenic bacteria. Contrary to our expectation, however, there were no significant differences in the diversity of the URT microbiota between children with and without nursery attendance. This may be because the characteristics of the environmental factors were not significantly different between these 2 groups. On the other hand, in the cluster analysis, there was a significant difference between the two groups. The proportion of samples with a high abundance of bacteria commonly isolated from the respiratory tract in children with respiratory infections, such as *H. influenzae*, was higher in the samples from children with nursery attendance than in those from children without nursery attendance, suggesting that early nursery attendance may be associated with differences in the URT microbiota. Furthermore, the antibiotic therapy and the frequency of the development of URTI were also associated with differences in the URT microbiota. Nursery attendance and the environmental factors related to nursery attendance may be associated with differences in the URT flora in young children.

Nursery attendance increases the risk of the development of URTI in children (16), and viral infection contributes to differences in the URT microbiota (17). *H. influenzae* was overrepresented in children with viral infection in a previous study (17). In our study, the proportion of the samples classified into the *H. influenzae*-dominated cluster was high in those collected from children with a high incidence of URTI (≥3 episodes). However, there was no significant difference in the proportion of children with a history of total of ≥3 URTI episodes during the investigation period by nursery attendance. Although the reason for this result is unclear, we speculated that household transmission of various viruses might frequently happen, even in children without nursey attendance, as previous studies indicated that children were more susceptible than adults to the secondary transmission of viruses within households (18–20).

We also found no significant difference in the proportion of children receiving antibiotics between those with and without nursery attendance. However, the proportion of children treated with antibiotics before 12 months old was significantly higher in the nursery attendance group than in the non-nursery attendance group (Table 2). In our study, the proportion of samples classified as the *C. propinquum*-dominated cluster was significantly higher in children aged ≥6 months old without nursery attendance than in those with nursery attendance. *Corynebacterium* species are one of the main bacteria forming the URT microbiota in young children (21, 22). In a previous study, antibiotic therapy prior to the collection of URT sample was associated with lower amounts of *Corynebacterium* and a greater abundance of *Haemophilus* and *Moraxella* genera (15). Our data suggested that the use of antibiotics rather than the development of URTI in younger children may primarily influence the difference in the distribution of the clusters between children with and without nursery attendance.

The role of *Moraxella* in respiratory health is controversial. It has been reported that two distinct *Moraxella*-dominated microbiota profiles exist in young children: one highly suggestive of *M. catarrhalis* and the other corresponding to a *M. lincolnii* (22). *M. lincolnii* is a typical commensal bacterium from the URT, whereas the predominance of *M. catarrhalis* in the URT microbiota is associated with ARI episodes and the antibiotics use (4, 15, 22, 23). In this study, there were few samples classified into the *M. lincolnii*-dominated cluster (data not shown), whereas many samples were classified into the *M. catarrhalis*-dominated cluster. This result may be because most eligible children had received antibiotics before the latest sampling. In the identification of *Moraxella* species, the clone library analysis performed in this study may be more useful than next-generation sequencing, which is often insufficient for the identification of bacterium at the species level.

In this study, the proportion of the samples classified into the *H. influenzae*-dominated cluster was significantly higher in the samples from children using antibiotics between the previous and present samplings than in those who had not been administered antibiotics, whereas the opposite tendency was observed in the proportion of the samples classified into the *M. catarrhalis*-dominated cluster (Figures 3B, 4B). A previous study indicated that the use of antibiotics was associated with a higher abundance of both bacteria in the URT microbiota (15). On the other hand, another previous culture-based study indicated that colonization with *M. catarrhalis* in URT was not associated with the use of antibiotics (24). Given the results of the previous studies, as the association between the predominance of *M. catarrhalis* in the URT microbiota and antibiotic use seems to be controversial, *H. influenzae* may tend to be more predominant than *M. catarrhalis* in the URT microbiota of children who have received antibiotics.

In this study, the frequency of URTI episodes before sampling was also associated with differences in the URT microbiota. In the samples from children with a high frequency (≥3 times after first sampling) of URTI episodes, the proportion of the samples classified into the *H. influenzae*-dominated cluster was significantly higher than those from children with a low frequency of the development of URTI. The opposite result was observed in the proportion of the samples classified into the *M. catarrhalis*-dominated cluster. This result was similar to that obtained when comparing samples from children with and without receiving antibiotic therapy. A previous study indicated that viral infection was an important factor that modulates bacterial diversity in the URT (17). However, which pathogenic bacteria predominate in the URT microbiota of children with URTI episodes is unclear at present. Given the present findings, *H. influenzae* may tend to be more predominant in the URT microbiota of children with URTI.

This study was associated with some limitations. First, the study population was relatively small, which could have affected the accuracy of the statistical analysis. Second, some analyses were performed by using multiple samples collected from the same child. Although these samples were not always classified as the same cluster, this factor may raise some concerns regarding reproducibility and validity. Third, the clone library analysis has more technical limitations than the metagenome sequencing analysis. The clone library analysis has an advantage in that it can precisely identify bacteria at the species level because of the relatively long length of sequences compared to next-generation sequencing (25, 26). As we wish to identify bacteria at the species level, a clone library analysis was performed in this study. Finally, it is impossible to amplify all bacterial 16S rRNA genes completely. Although the sensitivity of the primers for the bacterial species used in this study was approximately 92%, not all pathogenic bacteria were able to be detected by using these primers (27).

In conclusion, nursery attendance, the use of antibiotics and recurrent URTI in young children may influence differences in the URT microbiota. Children with nursery attendance tended to receive antibiotics during infancy compared to those without nursery attendance, and antibiotic use was associated with an increase in pathogenic bacteria, such as *H. influenzae*. Further studies are called for investigating the impact of the increase of pathogenic bacteria in the URT microbiota in children.

## Data Availability

The raw data supporting the conclusions of this article will be made available by the authors, without undue reservation.
